# Supplementary material for: Stacking Model‐Based Classifiers for Dealing With Multiple Sets of Noisy Labels
Source: Biom J. 2025 Mar 12;67(2):e70042. doi: 10.1002/bimj.70042 (PMC11898607; doi:10.1002/bimj.70042)
Supplement: Supplementary file 1 — Supporting Information [file BIMJ-67-e70042-s001.zip › density-based-ensemble-model-main/competitors_real_data/netlab-master/netlab-master/help/demgauss.htm]

Netlab Reference Manual demgauss


# demgauss

## Purpose

Demonstrate sampling from Gaussian distributions.

## Synopsis

```
demgauss
```

## Description

`demgauss` provides a simple illustration of the generation of
data from Gaussian distributions. It first samples from a
one-dimensional distribution using `randn`, and then plots a
normalized histogram estimate of the distribution using `histp`
together with the true density calculated using `gauss`.

`demgauss` then demonstrates sampling from a Gaussian distribution
in two dimensions. It creates a mean vector and a covariance matrix,
and then plots contours of constant density using the function
`gauss`. A sample of points drawn from this distribution, obtained
using the function `gsamp`, is then superimposed on the contours.

## See Also

`gauss`, `gsamp`, `histp`

---

**Pages:**
Index


---

Copyright (c) Ian T Nabney (1996-9)
